# Recent Advances in Nanomaterials Used for Wearable Electronics

**DOI:** 10.3390/mi14030603

**Published:** 2023-03-05

**Authors:** Minye Yang, Zhilu Ye, Yichong Ren, Mohamed Farhat, Pai-Yen Chen

**Affiliations:** 1 Department of Electrical and Computer Engineering, University of Illinois at Chicago, Chicago, IL 60607, USA; 2 Division of Computer, Electrical and Mathematical Sciences and Engineering, King Abdullah University of Science and Technology (KAUST), Thuwal 23955-6900, Saudi Arabia

**Keywords:** nanomaterials, flexible substrates, soft conductors, wearable sensors, healthcare monitoring

## Abstract

In recent decades, thriving Internet of Things (IoT) technology has had a profound impact on people’s lifestyles through extensive information interaction between humans and intelligent devices. One promising application of IoT is the continuous, real-time monitoring and analysis of body or environmental information by devices worn on or implanted inside the body. This research area, commonly referred to as wearable electronics or wearables, represents a new and rapidly expanding interdisciplinary field. Wearable electronics are devices with specific electronic functions that must be flexible and stretchable. Various novel materials have been proposed in recent years to meet the technical challenges posed by this field, which exhibit significant potential for use in different wearable applications. This article reviews recent progress in the development of emerging nanomaterial-based wearable electronics, with a specific focus on their flexible substrates, conductors, and transducers. Additionally, we discuss the current state-of-the-art applications of nanomaterial-based wearable electronics and provide an outlook on future research directions in this field.

## 1. Introduction

The rapid proliferation of Internet of Things (IoT) applications, enabled by the advancement of 5G and beyond technologies, has significantly transformed people’s way of life in recent years by connecting physical objects, such as sensors and memories, for data and information collection and exchange [1,2]. The adoption of wearable electronics is an area of remarkable growth, allowing individuals to access and interact with the IoT in novel and convenient ways [3,4]. The pliability and softness of wearable electronics make them suitable for use as an accessory or integrated into clothing, enabling the continuous and real-time exchange of information between devices and users while on the move. This degree of performance would not be feasible with traditional electronic technologies, which are typically composed of rigid and bulky materials.

The development of wearable electronics has been propelled by advancements in technology, particularly in the domains of microelectronics [5], material science [6], and sensors and actuators [7,8]. These advancements have resulted in a broad range of practical applications, including smart watches [9,10], fitness trackers [11,12], and augmented reality (AR) glasses [13,14,15]. In recent years, wearables have demonstrated outstanding promise in healthcare monitoring applications [16,17,18], necessitating that they be flexible, stretchable, twistable, and/or biocompatible for attachment to the human epidermis or for implantation within the body. Tremendous research efforts have been focused on advanced materials that meet these challenges, with nanomaterials drawing particular attention due to their distinctive mechanical, chemical, and biological properties at the nanoscale. To date, a range of nanomaterials, including silicon nanomembranes [19,20], graphene [21], carbon nanotubes [22,23], liquid metal [24,25], and other chemical/organic nanomaterials [26,27], have been implemented in various wearable scenarios.

To ensure that wearable devices are completely compatible with the curved surface of the human epidermis, both the electronic component and the substrate must be flexible and stretchable, necessitating the use of nanomaterials with differing mechanical and electrical properties [28]. Electrical performance is not a major consideration for the substrate, but high air permeability and heat dissipation are crucial because the substrate may be in direct contact with the skin. Materials such as styrene–ethylene–butylene–styrene (SEBS) thermoplastic elastomer [29], polydimethylsiloxane (PDMS) [30], and polyimide (PI) [31,32] have shown great potential as soft platforms for wearables. On the other hand, the materials used in building electronic components, such as conductors and transducers, must have adequate electrical conductivity and other electromechanical properties (e.g., piezoelectricity and photovoltaics) to perform the desired functions of wearable devices. Examples include silver nanowires [20], liquid metal [33], soft ferroelectric materials [34], and temperature-sensitive materials [35], among many others.

In addition to wearable devices that operate on the surface of the skin or clothing, another class of wearable electronics known as implantable devices is placed inside the human body to monitor vital signs such as intracranial pressure [36], intestinal pressure [37], and intraocular pressure [38]. These devices have more stringent requirements for materials, particularly for biocompatibility, as the implants must not cause any unwanted inflammation. The materials used for these devices must also have a high tolerance to the influence of human tissues, ensuring that the implants maintain their performance after implantation. Some research groups have further investigated biodegradable materials for implants [39,40], which can safely degrade in the body after their intended use, reducing the number of necessary surgeries for implantation, maintenance, or replacement of the devices and lowering the risk of complications such as inflammation or hemorrhage.

This review article centers on the utilization of nanomaterials in wearable electronics, including their use as substrate materials to support electronic systems, conductive materials to connect different electronic components, and functional materials for translation of physical quantities to electrical signals. We also examine recent advancements in nanomaterial-enabled wearable electronics applications and offer an outlook on their potential future.

## 2. Nanomaterial-Enabled Wearable Electronics

### 2.1. Flexible and Biocompatible Substrates

**Figure 1:**
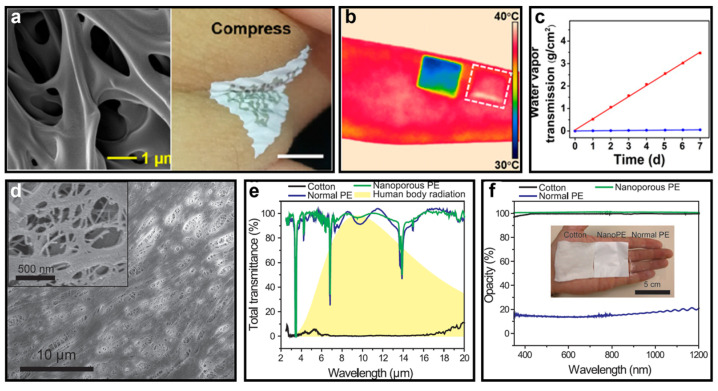
(**a**) Microscopic and macroscopic images of a nanoporous SEBS structure as a substrate attached to human epidermis. (**b**) Comparison of the passive cooling function of nanoporous and nonporous SEBS substrates. (**c**) The water vapor transmission of nanoporous SEBS (red) and nonporous SEBS (blue). ((**a**–**c**) Reprinted with permission from Ref. [47], Copyright 2019 National Academy of Sciences.) (**d**) Picture of nanoPE obtained from SEM. (**e**) Transmissions of body radiation of different material-based substrates. (**f**) Opacity under visible light of different material-based substrates. ((**d**–**f**) Reprinted with permission from Ref. [48], Copyright 2016 The American Association for the Advancement of Science.).

The substrate is an essential component in wearable electronic systems as it serves as the platform for operation and comes into direct contact with the skin. Thus, the materials used for flexible substrates should possess multifunctional properties to ensure biocompatibility, such as passive cooling [41], high breathability [42], waterproofing [43], recyclability [44], and minimal impact on electronic systems. One practical approach to achieve these objectives is the use of nanoporous materials made from biocompatible natural or synthetic polymers, such as gelatin [45], poly(glycerol sebacate) (PGS) [46], SEBS [29], and polyethylene (PE) [41]. For example, Figure 1a–c depict a multiscale porous SEBS-based substrate proposed by Yadong Xu et al. that meets these requirements [47]. In contrast to conventional SEBS substrates, this novel porous SEBS substrate is impregnated with multiscale nanopores that provide not only the usual properties of a flexible substrate but also high sunlight reflectance and low reflectance for body radiation (IR), allowing for passive cooling without energy consumption. The porous SEBS is produced using a simple, inexpensive, and scalable phase-separation-based process, which involves preparing a solution of SEBS and isopropyl alcohol (IPA) in chloroform, coating it onto an aluminum foil, and evaporating the volatile chloroform to create phase separation of the IPA from the SEBS, forming nano-/microscale droplets. The subsequent evaporation of these droplets results in a porous SEBS substrate with pores of varying sizes. It is noteworthy that the evaporation of the organic solvents must proceed under specific conditions to ensure that the fabrication process is harmless to the environment.

Figure 1b clearly illustrates the impressive passive cooling capabilities of the proposed porous SEBS compared to a normal flexible substrate. The epidermis covered by the porous SEBS is 6 °C cooler than that covered by the nonporous SEBS. Furthermore, the porous SEBS may have a high water vapor transmission rate due to the interconnected hierarchical pores presented in the substrate, as shown in Figure 1c (red line for the porous substrate and blue line for the nonporous substrate). This multifunctional porous SEBS, when used as a substrate for wearable electronics, can significantly improve user comfort and reduce the risk of inflammation due to sweat accumulation. In addition to its exceptional biocompatibility, the porous SEBS substrate can also support bioelectronic devices, such as pressure/strain sensors [29] and electromyography (EMG) sensors [49], through the spray printing of conductive materials such as silver nanowires (Ag NWs), which exhibit excellent mechanical compliance and electrical conductivity when applied to a porous SEBS.

Po-Chun Hsu and his colleagues proposed another promising nanomaterial as a substrate for on-skin electronics that can provide similar biocompatibility [48]. This material is a textile-like porous polyethylene with pore sizes ranging from 50 nm to 1000 nm, making it opaque to visible light and transparent to body radiation due to the distribution of pore sizes. Figure 1e and the inset of Figure 1f show a scanning electron microscopic (SEM) photograph of the nanoporous polyethylene (nanoPE) textile and its macroscopic structure, respectively. Additionally, Figure 1e,f demonstrate the ability of nanoPE to transmit body radiation while blocking visible light. These unique characteristics make the nanoPE textile an ideal candidate for making clothes-like substrates for wearables with excellent thermal management and passive cooling. Measurement results have shown that skin covered by nanoPE textile can be cooled by 2.7 °C compared to skin covered by normal cotton. Furthermore, the nanoPE textile can also exhibit excellent air permeability and waterproofing after specific chemical modification. Although the nanoPE textile may not have high stretchability and ductility, it can be processed into garments with relatively fixed shapes that can accommodate complex electronic systems.

**Figure 2:**
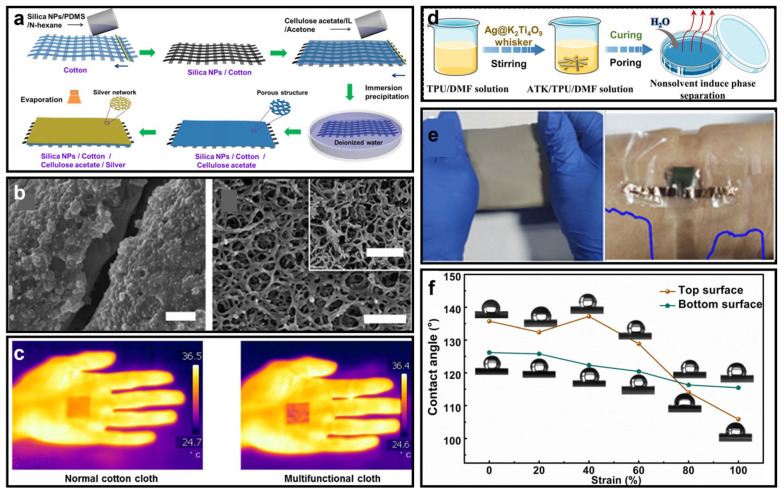
(**a**) Fabrication process of silica nanoparticle PDMS/nanoporous cellulose acetate film. (**b**) Microscopic photographs of the silica nanoparticle PDMS and the nanoporous cellulose acetate; the three scale bars are 10 µm, 2 µm, and 1 µm, respectively. (**c**) Passive cooling feature of the proposed hybrid material ((**a**–**c**) reprinted with permission from Ref. [52], Copyright 2018 American Chemical Society). (**d**) Fabrication process of the nanoporous PU substrate. (**e**) Photograph showing the stretchability of the nanoporous PU. (**f**) Waterproofness of the nanoporous PU under different strains ((**d**–**f**) are reprinted with permission from Ref. [53], Copyright 2022 Royal Society of Chemistry).

In addition to the two representative nanomaterials used for the substrate in a wearable electronic system, many other flexible materials have emerged in recent years, enabled by porous polymers with excellent mechanical properties and outstanding appliances for the spray painting of electronics, such as porous PDMS [50] and porous polyurethane (PU) [51]. Figure 2a illustrates the fabrication process of a nanoporous PDMS-based flexible substrate [52]. This hybrid material is made by integrating a silica nanoparticle/PDMS and nanoporous cellulose acetate layer on two sides of a cotton fabric, which can achieve excellent waterproofing and high air permeability. The microscopic image of the substrate presented in Figure 2b clearly shows the porous structure with pore sizes of approximately 500 nm. The passive cooling capability is depicted in Figure 2c, where a 2 °C temperature drop can be achieved by covering such a nanoporous PDMS-based substrate on the epidermis. It is worth noting that the nanoporous cellulose acetate layer introduces the passive cooling feature, while the nanoparticle/PDMS layer functioning as a superhydrophobic layer provides excellent waterproofness. Furthermore, a breathable, flexible substrate made of thermoplastic PU film proposed by Ziwei Chen et al. [53] is shown in Figure 2d. Their prepared porous membrane possesses a graded pore size distribution and can therefore exhibit high air permeability. At the same time, through integrating with Ag@K_2_Ti_4_O_9_, this substrate has enhanced sensitivity as a piezoresistive substrate. Figure 2e depicts a macroscopic photograph of the fabricated nanoporous PU film while stretched and attached to the epidermis for human–machine interactions, which shows its good stretchability. Additionally, this PU-based flexible substrate can achieve desired waterproofness, as shown in Figure 2f, with a contact angle of 140°. Table 1 presents a comparison of the four mentioned nanoporous-based substrates.

**Figure 3:**
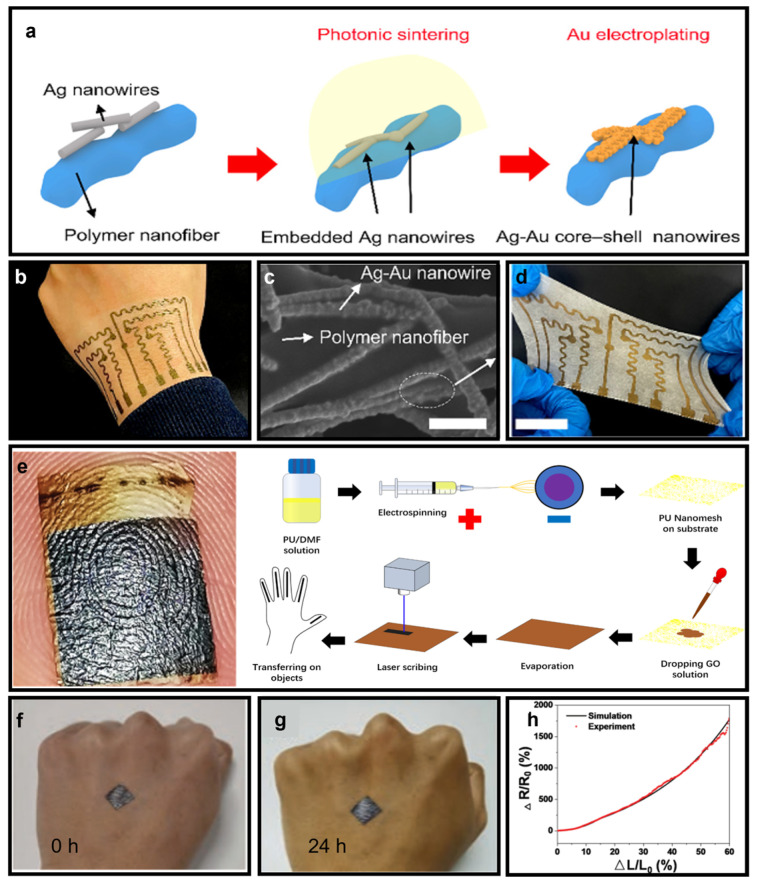
(**a**) Fabrication process of an Ag–Au nanowire network (AANN). (**b**) Photograph of an AANN substrate when attached to human epidermis (**c**) Microstructure of an AANN. (**d**) Stretchability of an AANN substrate. ((**a**–**d**) Reprinted with permission from Ref. [54], Copyright 2022 Published by Elsevier Ltd.) (**e**) Photograph of LSG/PU substrate when attached to human epidermis and its fabrication process. (**f**,**g**) Photographs of an LSG/PU substrate attached to epidermis for different time intervals. (**h**) Tensile property of an LSG/PU substrate ((**e**–**h**) reprinted with permission from Ref. [55], Copyright 2021 Wiley-VCH GmbH.).

In addition to porous-structure-based substrates, nanomesh-based substrates have also been developed that are ideal for supporting wearable electronics. While the former substrate has been found to exhibit suboptimal durability and electrical performance, the latter can effectively address these issues. Wooseong Jeong and his group proposed an Ag–Au nanowire network (AANN) synthesized with a polymer nanomesh substrate, as shown in Figure 3a–d [54]. The Ag nanowires are attached to the polymer nanofiber, and upon photonic sintering, Au is electroplated onto the Ag nanowires, forming Ag–Au core–shell nanowires. To fabricate this substrate, TPU elastomer solution is first diluted with a mixture of methyl ethyl ketone solvent and dimethylformamide (DMF) by stirring for six hours. The resulting solution is then placed in an electrospinning system with 15 kV voltage applied and ejected at 1.5 mL/h to obtain TPU elastomer nanofiber mesh structures. The Ag nanowire dispersion in ethanol is subsequently spray-coated onto the nanofiber mesh. This AANN-integrated substrate is essentially biocompatible, stretchable, and ultradurable, as evidenced by the photograph of the substrate attached to human epidermis and the microscopic SEM picture presented in Figure 3b and Figure 3c, respectively. Figure 3d is a demonstration of the impressive stretchability of this AANN substrate. However, the electrospun nanomesh-based substrate may be expensive to manufacture due to the use of Au to ensure biocompatibility. Yancong Qiao and his collaborators proposed a laser-scribe-graphene (LSG)/PU-based nanomesh-type e-skin (Figure 3e), which may significantly lower the cost of fabrication [55]. This substrate is ultralightweight and possesses good air permeability and fine conformability. The PU nanomesh has a low melting point and therefore cannot support the chemical vapor deposition of graphene, so a laser-scribed fabrication process is adopted, as depicted in Figure 3e. The graphene oxide dispersion is first mixed with tetrahydrofuran and poured onto the PU nanomesh. After full evaporation, the nanomesh is embedded into the graphene oxide film. In addition to its good gas permeability and comfortability, Figure 3f,g also demonstrate its remarkable durability, as the device remains unchanged even after being worn on a hand for different intervals of time. Since the substrate exhibits exceptional tensile properties, as shown in Figure 3h, it may have a bright future in pressure sensing applications. Both of these examples of nanomesh-based substrates have demonstrated the immense potential of this structure for use as a substrate for wearable electronics.

### 2.2. Soft Nanoconductors for Wearables

**Figure 4:**
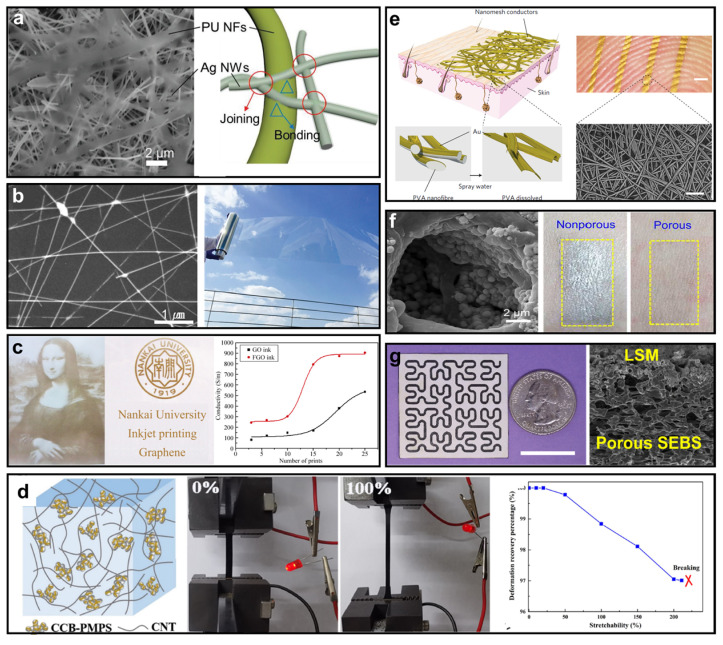
(**a**) Microscopic photograph and diagram of the conducting network of Ag NWs and PU NFs (reprinted with permission from Ref. [58], Copyright 2019 John Wiley & Sons, Inc.). (**b**) Microscopic photograph of Ag NWs/PEDOT:PSS and its transparent nature (reprinted with permission from Ref. [59], Copyright 2015 Royal Society of Chemistry). (**c**) Graphene nanoink-fabricated patterns and their conductivity (reprinted with permission from Ref. [60], Copyright 2011 Springer Nature). (**d**) Structure of the carbon nanotube–propyl methacrylate–conductive-carbon back (CNT-PMPS-CCB), a demonstration of its stretchability, and its recovery rate under different stretchability conditions (reprinted with permission from Ref. [61], Copyright 2020 Elsevier Ltd.). (**e**) Diagram of Au nanomeshes, photographs of the conductor attached to the epidermis, and an SEM image (reprinted with permission from Ref. [62], Copyright 2017 Springer Nature). (**f**) Porous liquid metal in under a microscope and comparison of liquid leakage between the porous and nonporous liquid metal (reprinted with permission from Ref. [63], Copyright 2023 The American Association for the Advancement of Science). (**g**) MoO_2_-enabled Hilbert serpentine pattern and its SEM image (reprinted with permission from Ref. [64], Copyright 2022 The American Association for the Advancement of Science).

If the substrate of a wearable electronic system can be considered as the foundation of a skyscraper, then the conductor can be seen as the mainframe of the skyscraper. This is because the conductive materials used in wearable electronics must not only be flexible but also have favorable electrical conductivities to ensure that the function of the electronic systems does not degrade compared to those composed of traditional rigid conductors, such as copper or gold. Ag NW is one of the most popular nanomaterials used for flexible conductors due to its high conductivity and ease of fabrication through methods such as spray painting, coating, and inkjet printing [56,57]. Zhi Jiang and his group proposed an extraordinary example of nanomesh-type elastic conductors, as shown in Figure 4a [58]. This porous nanomesh-type conductor is made of two layer-by-layer nanofibers (NFs) or nanowires (NWs) produced through interfacial hydrogen bonding. High conductivity and stretchability are achieved by adhering the highly conductive Ag NWs to the stretchable PU NFs, as illustrated in Figure 4a. Moreover, this conductor exhibits good cyclic durability, facilitating a prolonged wearable lifetime. It has been reported that their proposed flexible conductors exhibit a conductivity near 9190 S/cm, with stretchability of up to 310%. The resistance of the conductor increases by up to 82% after 1000 stretch/release cycles at up to 70% tensile strain. Notably, they have realistically created an electrode with a nanomesh-type conductor that is 2.5 mm wide and 20 mm long, the resistance of which can be less than 1.5 Ω. Such performance is very close to that of traditional metallic materials, demonstrating its considerable potential for use in connecting electronic systems in wearable devices.

In addition to the nanomesh-type conductors made by adhesion between Ag NWs and PU NFs, another flexible conductor enabled by Ag NWs and poly(3,4-ethylenedioxythiophene):poly(styrenesulfonate) (PEDOT:PSS) was proposed by Seyul Kim [59]. PEDOT:PSS is a well-known conducting polymer that can enhance the conductivity of metallic nanowires by functioning as an electrical bridge between separated nanowires [65]. However, the traditional fabrication process of Ag NWs/PEDOT:PSS may result in reduced conductivity due to the rough surface of PEDOT:PSS on top of Ag NWs and the necessary additive binders that prevent the aggregation of Ag NWs, which may, in turn, disrupt electron transfer from PEDOT:PSS to Ag NWs [66,67]. To address these issues, Seyul Kim et al. proposed a one-step coating without any post treatment, significantly enhancing the conductivity. Figure 4b shows the microstructure of Ag NWs/PEDOT:PSS and a macroscopic photograph; Ag NWs/PEDOT:PSS are coated on a transparent polyethylene terephthalate (PET) film. The measured sheet resistance is reported to be 10.8 Ω/sq. This conductor is also transparent in visible light, providing additional benefits for wearable electronics incorporated into clothing, as they do not compromise the integrity and beauty of clothes. Although these two types of Ag NW-based flexible conductors may have advantages such as remarkable durability or transparency, they both suffer from the relatively high cost of Ag NWs and unstable conductivity due to the non-uniform distribution of Ag NWs.

Single-layered graphene oxide (GO) or few-layered graphene oxide (FGO) conducting films are promising alternatives for use in flexible conductors due to the superb electrical properties of graphene [68,69,70]. Lu Huang et al. proposed an extremely easy fabrication process for GO or FGO conductors using a cheap commercial inkjet printer [60]. The use of inkjet printing to make soft electronics has several advantages, including compatibility with different substrates, contactless and mask-free patterning, and vacuum-free processing [71]. GO and FGO solutions were obtained by oxidizing graphite with a modified Hummers method, which, after proper preparation for a certain viscosity, were then injected into a cleaned ink cartridge. The GO and FGO were printed onto soft substrates, such as paper, PET, and PI, following predesigned patterns. The printed pattern and measured conductivity are depicted in Figure 4c. The conductivity can be significantly improved by repeating prints or using FGO ink. This may be attributed to the multiple prints leading to a cascaded connection among different prints, effectively reducing the surface impedance of the conducting film. Compared to previous Ag NW-based conductors, GO and FGO have much simpler fabrication processes, although they have lower conductivity. Additionally, although conductivity can be improved by multiple prints, the alignment among different prints can be a challenge. The authors demonstrated the application of this conductor as a flexible electrode for H_2_O_2_ sensing, which does not have rigorous requirements for conductivity. Therefore, these GO and FGO conducting materials may have a bright future, e.g., as electrodes for wearable electrocardiogram (ECG) and EMG sensors.

Furthermore, the utilization of the carbon nanomaterial family has attracted significant research interest. Figure 4d demonstrates a hybrid carbon nanotube–propyl methacrylate–conductive-carbon back (CNT-PMPS-CCB) conductor proposed by Pan Song et al. [61]. This conductor may possess excellent mechanical properties, with an elongation at break of 211% and a tensile strength of 4.5 MPa, in addition to a high electrical conductivity that can reach 248.8 S/m. The fabrication process begins with the preparation of CCB-PMPS. CCB and MPS monomers are first placed in dry toluene and ultrasonicated for 30 min. The mixture is washed three times with toluene to obtain self-polymerized PMPS, which is highly soluble in toluene. After drying in a vacuum oven, the CCB-PMPS is prepared. Next, CNTs containing hydroxyl groups react with the prepared CCB-PMPS to obtain the CCB-P-CNT hybrid filler. The first panel of Figure 4d clearly shows the structure of the synergistic dispersion of the CNTs via CCB-PMPS. The conductor’s great mechanical stretchability is demonstrated in Figure 4d, where the light-emitting diode connected to the conductor can light up when 100% strain is applied to it. Additionally, the conductor can have a good recovery rate within a certain range of applied strain, as shown in the last panel of Figure 4d. Furthermore, the authors proposed utilizing the conductor’s high strain sensitivity and excellent deformation recovery performance for sensing applications.

In addition to the nascent nanomaterials used for soft and pliable conductors, traditional metallic conductors that employ novel fabrication processes to attain both inflammation-free and stretchability requisites have also captured the interest of numerous researchers [72,73]. A noteworthy example of this is the Au nanomeshes introduced by Akihito Miyamoto and his colleagues [62]. Nanofibers with diameters ranging from 300 nm to 500 nm were produced from a polyvinyl alcohol (PVA) solution and configured into a mesh-like sheet. Subsequently, an Au layer was deposited onto the sheet using a shadow mask. This nanomesh conductor could be directly placed on the epidermis or other soft substrates, and the PVA nanofibers could be easily removed by spraying water. Figure 4e lucidly illustrates the conductor’s structure and its macro-/microscopic photographs. The fabricated nanomesh conductor proposed in this study, which had a width of 2.5 mm and a length of 80 mm, might have a resistance close to 600 Ω, which is significantly superior to that of previous nanoconductors. Additionally, this Au nanomesh conductor demonstrates remarkable robustness against stretch/release cycles, as the conductance may remain unchanged within a 15% strain and display limited reduction within a 50% strain. It is quite surprising that even after 500 stretching cycles, the conductance may not undergo any substantial changes, suggesting that it has excellent robustness. Compared to GO and FGO conductors, the drawbacks of this Au nanomesh conductor might be its relatively complicated fabrication process that requires deposition and post treatment, as well as the high cost of Au.

Using a similar principle of direct utilization of metal conductors, liquid metal has piqued growing research interests as a flexible conductive material for next-generation skin-interfaced bioelectronics [24,25,33,74]. However, the primary challenge associated with liquid metal conductors is the difficulty of confining the liquid metal within a specific area serving as conductors and the inevitable leakage of the liquid metal during the deformation of the substrate. In Ref. [63], Yadong Xu and his colleagues proposed a porous liquid metal–elastomer composite as a soft conductor with high leakage resistance and antimicrobial properties. It concurrently possesses high conductivity conferred by the liquid metal and high resistance against deformations due to its porous structure. In this study, eutectic gallium–indium (EGaIn) liquid metal was selected, as it can provide the desired electrical conductivity, negligible vapor pressure, and nontoxicity [75]. Furthermore, EGaIn can also retain its electrical conductivity against deformations. The fabrication process begins with the sonication of EGaIn in 1-butanol to generate the liquid metal in micro/nanoscale particles. Subsequently, the particles are integrated into a bis(2-ethylhexyl) sulfosuccinate (BEHS)-modified epsilon polylysine (ε-PL) and PU solution in tetrahydrofuran (THF) to imbue the composite with antimicrobial properties. This precursor solution is then transferred onto an aluminum foil and dried. Since the volatile THF has a relatively low boiling point compared to 1-butanol, the complete evaporation of THF and 1-butanol in a separated phase eventually leads to the formation of porous EGaIn composites. As illustrated in the left panel of Figure 4f, EGaIn particles adhere to the surface of pores during the phase separation process and interconnect with one another to form conductive pathways via mechanical sintering. This results in high resistance against the leakage of liquid metal, as shown in the right panel of Figure 4f. Subsequently, the composite can be fashioned into different patterns by laser cutting and exhibits high leakage resistance. Nonetheless, some other liquid-metal-based conductors may require additional assistance to maintain a particular shape [76].

In addition to conventional metallic materials, numerous research endeavors have been devoted to emerging metal oxide conductors such as zinc oxide [77] and manganese dioxide (MnO_2_) [78], which possess captivating electrical conductivity, high chemical stability, high biocompatibility, and low magnetic susceptibility. Ganggang Zhao and his colleagues proposed laser-assisted, mask-free, scalable scribing of molybdenum dioxide (MoO_2_) (LSM), which not only exhibits the abovementioned superior traits but also employs a simple, low-cost, and powerful fabrication technique [64]. Figure 4g lucidly displays a Hilbert serpentine pattern produced by LSM for demonstration. The fabrication process begins with a spray coating of molybdenum chloride (MoCl_5_) onto a supporting substrate (e.g., rigid acrylic or flexible SEBS), which is annealed in the air. Subsequently, CO_2_ irradiation is employed to transform the precursors into MoO_2_ via a photothermal process. Although such laser-assisted fabrication techniques have been employed in, for instance, laser-scribed SiC, they may lead to undesired electrical conductivity [79,80]. On the contrary, the fabricated LSM conductor may have a mere 0.4 Ω/sq, which can outperform the nanoconductors based on Ag NWs and graphene due to its metallic nature. Compared to the previously described nanoconductors, this laser-scribed LSM may additionally possess the most precise fabrication process, with intermediate fabrication complexity and cost. Such performances can establish LSM as an ideal candidate for wearable electronics, as it significantly reduces energy loss in the system. Table 2 presents comparisons of the reviewed nanomaterial-based soft/flexible conductors.

### 2.3. Nanomaterial-Based Transducers

**Figure 5:**
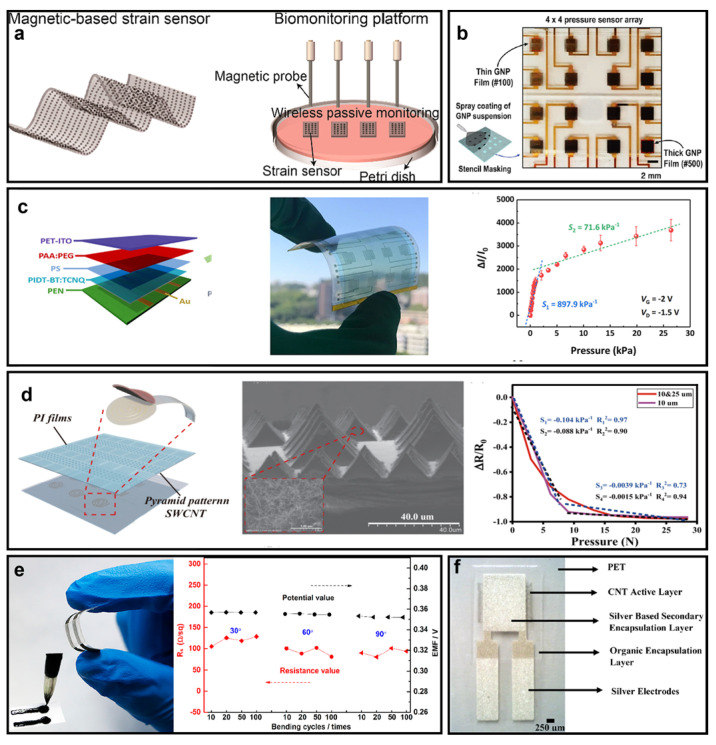
(**a**) A strain transducer based on (GelMA)/Fe_3_O_4_–magnetic hydrogel film (reprinted with permission from Ref. [86], Copyright 2022 American Chemical Society). (**b**) A pressure transducer array made of graphene nanoparticles (reprinted with permission from Ref. [87], Copyright 2021 Elsevier, Ltd.). (**c**) A pressure transducer made of organic field-effect transistors (reprinted with permission from Ref. [88], Copyright 2020 Elsevier Ltd.). (**d**) A pressure transducer built from carbon nanotubes (CNTs) with pyramid patterns (reprinted with permission from Ref. [89], Copyright 2022 Springer Nature). (**e**) A cadmium ion-selective membrane made of graphene nanosheet (reprinted with permission from Ref. [90], Copyright 2018 American Chemical Society). (**f**) A temperature transducer made of carbon nanotubes (reprinted with permission from Ref. [91], Copyright 2018 Elsevier, Ltd.).

Following the discussion on flexible substrates and conductors, we now shift our attention to the functional component of wearable electronics, namely the transducer. Transducers may be the most effective devices for converting desired physical quantities into electrical signals that can be leveraged for post-signal processing [81]. Examples of transducers encompass temperature [82,83], pressure/strain [84,85], and ion concentration transducers, among others. Recently, Qi Zhang et al. introduced a magnetic-hydrogel-based soft strain transducer that exploits the relative magnetic field changes of a gelatin methacrylate (GelMA)/Fe_3_O_4_ magnetic hydrogel film, as illustrated in Figure 5a [86]. This material exhibits excellent magnetic properties (12.74 emu/g), great biocompatibility, desired stability, and a very low Young’s modulus that renders it ultrasoft. The fabrication of this strain transducer entails preparation of GelMA, which is then dissolved in a phosphate-buffered saline (PBS) solution containing 1% photoinitiator for 10 min. The superparamagnetic iron oxide nanoparticles are subsequently added to the GelMA solution and sonicated for 30 min. A static magnetic field with a specific magnetic flux density is applied to make the nanoparticles move and attain thermal equilibrium. The magnetic hydrogel film is then obtained under UV light, which crosslinks the magnetic hydrogel with an ordered distribution of magnetic nanoparticles (MNPs). It is worth highlighting that the concentration of the GelMA is strictly selected to satisfy both a low Young’s modulus and sufficient crosslinking. When external strain is applied to the film, it exhibits varying magnetic fields that can be leveraged for use as a strain transducer. Remarkably, this transducer exhibits sensitivity towards strain variations, responding to bending as low as 50 µm. The authors found the transducer to be stable, with a prolonged lifetime and free from ionic interference. In summary, this magnetic-hydrogel-based soft strain transducer shows great potential for implementation in wearable electronics for monitoring of, e.g., ECG signals, respiratory regulation, and bone growth.

Similar to the soft strain transducer, the on-skin pressure transducer also holds promising application scenarios. Jong-Seok Kim and his colleagues proposed a nanomaterial-based flexible pressure transducer array, as depicted in Figure 5b, that can accurately detect applied pressure, even on uneven surface curvatures [87]. This pressure transducer array is fabricated using graphene nanoplatelets (GNPs), which are mixed in DMF to form a suspension. The suspension is then coated on a polyethylene naphthalate (PEN) substrate, which is deposited with platinum electrodes to create a 4 × 4 GNP transducer array. When a certain pressure is applied to this array, the GNP nanoparticles establish electrical connections instead of a random distribution without pressure applied. This leads to a decrease in the resistance of the array, thereby completing the process of translating the pressure information to an electrical signal. Moreover, since this flexible transducer is ultimately attached to an uneven surface, which may introduce a mismatch of piezoresistive response in the individual pressure transducer, the authors developed a specific calibration method to ameliorate the inaccuracy caused by this mismatch. This unique circuit design and calibration method make this on-skin pressure transducer the ideal candidate for monitoring environmental pressure changes. However, a significant drawback of this design is that the transducer array has a relatively large size, which is not desired for in vivo pressure monitoring scenarios, such as monitoring of intracranial pressure, intraocular pressure, and intestinal pressure. Thus, we introduce two additional low-profile and miniaturized pressure sensors. Ziyang Liu and his team proposed a polyelectrolyte-gated organic field-effect (OFET) pressure sensor, as shown in Figure 5c [88]. The OFET is produced on a flexible plastic substrate, polyethylene naphthalate (PEN), on which the drain and source electrodes are located. The semiconductor layer comes in direct contact with the electrodes through spin coating, followed by the application of an insulating polystyrene (PS) passivation layer and a dielectric layer made of polyelectrolyte, which is also spin-coated onto the semiconductor layer. A PI tape with adjustable thickness is attached to the PEN substrate to support the structure. Finally, a 50 µm-thick PET film coated by indium tin oxide (ITO) serves as the suspended gate electrode for pressure sensing. In the inset of Figure 5c, we show a practical photograph of the fabricated sensor array, which is essentially compact and flexible. Moreover, the pressure sensitivity displayed in the right panel of Figure 5c illustrates its remarkable ability to detect human movements and gestures. Another example of a pressure sensor made of carbon nanotubes/polyimide (CNTs/PI) is presented in Figure 5d, which was proposed by Fuqin Sun and his colleagues [89]. The pyramid pattern of CNT/PI, shown in the inset of Figure 5d results in excellent sensitivity, with a resolution of 0.0015 kPa^−1^. The CNT/PI layer serves as the sensitive layer, while PET films with Ag electrodes function as the substrate, forming a relatively simple structure with greater feasibility. The fabrication process begins by preparing a Si mold with different micropyramids, followed by adding the CNTs in N-dimethylformamide (DMF) for ultrasonication dispersion. Following the preparation of the CNT/PI films with a pyramid pattern, a sealant is used to attach these films to the PET substrate with Ag electrodes. In comparison with the previous two nanomaterial-based pressure sensors, this CNT/PI-based pressure sensor has a simpler structure, higher sensitivity, and an easier fabrication process, making it suitable for various applications in on-skin electronics.

In addition to strain and pressure, information on ion concentrations such as Na^+^, K^+^ [92,93], glucose [94], and pH levels [95,96,97] in sweat or saliva are also crucial for healthcare monitoring. Therefore, wearable potentiometric transducers that can effectively detect changes in these indicators have attracted significant research attention. Among many others, one particular wearable potentiometric transducer, as shown in Figure 5e, was proposed by Chengmei Jiang and their group [90]. This transducer is constructed on a PET substrate by printing graphene nanosheet (GN)-based ink, a cadmium ion-selective electrode (Cd^+^-ISE), and the reference electrode. As a result, when the cadmium ions pass through the ion-selective electrode, the relative electrical potential between the Cd^+^-ISE and the reference electrode changes, achieving the ion detection function. Furthermore, this potentiometric sensor has high durability against bending and stretching cycles, as seen in Figure 5e; the conductivity and electromotive force under the same ion concentration remain almost unchanged. It is evident that this all-writing potentiometric transducer is cost-effective, as it does not require a sophisticated fabrication process and has high sensitivity due to the high conductivity and fast electron-transfer kinetics of GN. Moreover, the Cd^+^-ISE can be readily replaced by other selective electrodes sensitive to other ions, glucose, and pH to efficiently detect physiological indices. However, this all-writing fabrication technique may have a significant disadvantage: it cannot support precise fabrication compared to laser-induced fabrication or spray coating.

Body temperature is critical for clinical diagnosis, as it directly reflects the patient’s health condition [98]. Therefore, wearables that continuously acquire and analyze temperature information are important. Figure 5f demonstrates a negative temperature coefficient (NTC) thermistor based on carbon nanotubes (CNTs) that can translate temperature variations into resistance changes [91]. CNTs have been reported to have great NTCs, making them an ideal alternative to traditional NTC thermistors such as manganese-nickel-oxide, exhibiting significant changes in resistivity due to stoichiometry during the fabrication process. CNT temperature transducers are fabricated by screen printing on a PET film, representing a high-precision fabrication process that is low-cost and highly compatible with other soft substrates.

## 3. Applications of Nanomaterial-Enabled Wearable Electronics

**Figure 6:**
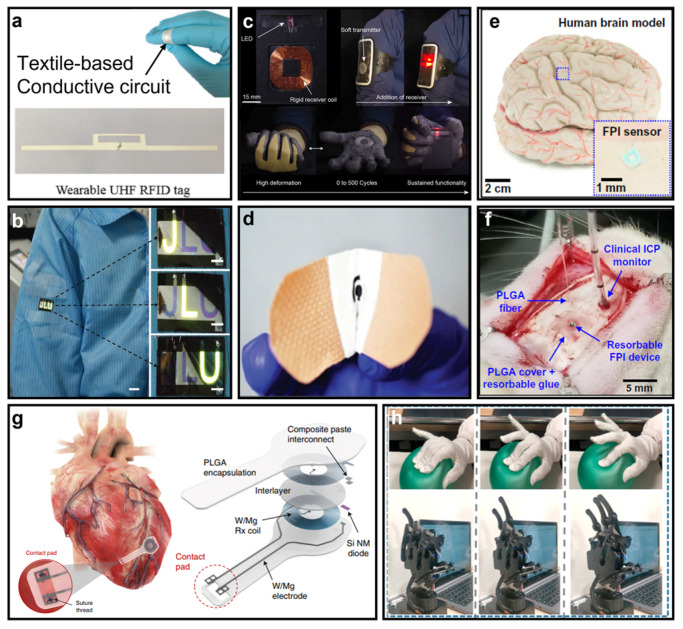
(**a**) Ultraviolet-curable nanomaterial-based on-skin RFID (reprinted with permission from Ref. [100], Copyright 2019 American Chemical Society). (**b**) Nanomaterial-enabled wearable display (reprinted with permission from Ref. [103], Copyright 2020 John Wiley & Sons, Inc.). (**c**) Wearable wireless power transfer enabled by a hybrid of magnetic colloidal and soft elastomers (reprinted with permission from Ref. [106], Copyright 2020 American Chemical Society). (**d**) Smart bandage achieved by soft material (reprinted with permission from Ref. [108], Copyright 2018 John Wiley & Sons, Inc.). (**e**) A bioresorbable nano-pressure sensor made with a Fabry–Pérot interferometer (FPI) structure attached to a human brain model. (**f**) Photograph of an FPI sensor implanted in the intracranial space of a rat. ((**e**,**f**) Reprinted with permission from Ref. [36], Copyright 2019 The American Association for the Advancement of Science). (**g**) A nanomaterial-based peacemaker that is free of leads and batteries (reprinted with permission from Ref. [111], Copyright 2021 Springer Nature). (**h**) Human–machine interactions enabled by a nanomaterial-based pressure transducer (reprinted with permission from Ref. [112], Copyright 2020 American Chemical Society).

We have reviewed the fundamental components of wearable electronics, including the soft substrates, conductors, and sensors. Each segment of wearable electronics has unique characteristics, which together construct the functional system and are interesting in many application scenarios, such as radio-frequency identification (RFID) [99,100] (Figure 6a), wearable displays [101,102,103] (Figure 6b), wearable wireless power transfer [104,105,106] (Figure 6c), and healthcare monitoring [107,108] (Figure 6d). RFID is a well-developed technique for identification and authentication, e.g., for parking lots, door access control, and anticounterfeiting technology. Conventional RFIDs are typically used in a credit-card-like form and commonly made using printed circuit board (PCB) technologies, which are usually bulky and rigid [109]. This characteristic, in many scenarios, requires the user to intentionally carry a key card around to prepare for the identification or authentication process, which is not time-efficient or convenient. In sharp contrast, nanomaterial-based RFID can essentially be flexible to comply with the uneven curvature of the epidermis while providing the same functionality as a traditional RFID technology. A particular prototype of nanomaterial-based flexible RFID is shown in Figure 6a [100]. This class of RFID can even be made as a tattoo on the human body instead of being worn [110]. Therefore, one can effectively avoid scenarios in which forgetting a key card may lead to losing access.

Wearable displays are a critical area for on-skin electronics. Traditional display techniques such as liquid crystal displays (LCDs) [113] and light-emitting diodes (LEDs) [114] are constructed upon rigid substrates and therefore require undeformed operation platforms such as televisions, desktop monitors, and smartphone screens. Consequently, they are unsuitable to be worn on the body to visualize healthcare information such as body temperature, heart rate, and blood oxygen levels acquired by biosensors. The prototype of a wearable display made of nanofabrics shown in Figure 6b [103] may provide an opportunity to address this limitation, allowing physiological signals interrogated by a local wearable sensor to be directly displayed without being affected by movement, bending, and other deformations. Wearable displays may also be ideal candidates for next-generation smart devices, wherein screens can be integrated with clothing, separate from CPUs or GPUs, to provide convenience on the go and avoid the loss of valuable devices.

Figure 6c showcases a cutting-edge wearable wireless power transfer system based on a hybrid nanomaterial design [106]. While traditional near-field wireless power transfer has been useful for convenient charging of electronic devices [115], recent advancements in wearable healthcare monitoring systems necessitate an on-skin power supply. Battery-powered wearables may have reduced lifetimes and relatively large sizes and may potentially harm human bodies due to their chemical composition. Therefore, wireless energy supply is of paramount importance for on-skin devices. The wearable wireless power transfer system depicted in Figure 6c provides a means to wirelessly receive power from an external source, allowing for continuous operations of local electronic systems. Additionally, this innovative class of wearable wireless power transfer systems can harvest radio-frequency energies in free space, enabling the charging of smart devices that users carry with them [116].

Advanced bandages such as that depicted in Figure 6d [108] and other on-skin wound-healing systems may significantly benefit from the integration of emerging nanomaterials. Since smart bandages come in direct contact with the wound bed, they must adhere to stringent requirements of high biocompatibility and nontoxicity [117]. In comparison to traditional bandages, the aforementioned technology may enhance the process of wound healing by continuously monitoring several physiological indicators of the wound bed, such as temperature, humidity, and pH level, dispensing medication automatically to the focus of infection [118]. In contrast to conventional smart bandages that consist of integrated circuits and systems, nanomaterial-based smart bandages may possess exceptional flexibility and softness, thereby ensuring optimal patient comfort. This trait may render them ideal substitutes for wound- and injury-healing applications.

Implantable electronics are another critical area that can provide real-time and continuous monitoring of vital signals, which can significantly benefit clinical diagnosis, treatments, and surgical protocols. The Fabry–Pérot interferometer (FPI)-based optical sensor proposed by the group of John A. Rogers (Figure 6e,f) is bioresorbable and can be implanted in vivo [36]. This sensor can simultaneously monitor variations in temperature and pressure, as demonstrated in an in vivo experiment that monitored intracranial pressure and temperature. Figure 6f depicts the FPI optical sensor implanted into the intracranial space of a rat, with remarkable sensitivity performance; the sensor can be fully resorbed by human tissue after its lifetime without any adverse effects. The sensing principle of this device relies on the reflection coefficients of the FPI cavity, which consists of inorganic silicon nanomembranes and biopolymers such as poly(lactic-co-glycolic acid) (PLGA). This work demonstrates the possibility of achieving wireless, real-time, and continuous monitoring of vital signals, such as intracranial pressure and temperature, via implantable sensors. Cardiac pacemakers have also garnered significant research interest. For example, Figure 6g illustrates a bioresorbable cardiac pacemaker proposed by Yeon Sik Choi et al. [111] that is free of leads and batteries. This pacemaker is suitable for temporal peacemaking during surgical recovery, which commonly involves percutaneous leads and external power sources. The device can be fully resorbed when exposed to biofluids through metabolic action and hydrolysis, thereby avoiding the need for mandatory surgery to remove traditional pacemakers. Compared to the aforementioned sensing system, this pacemaker requires the anticipation of metal electrodes and coils for energy harvesting made of biocompatible tungsten-coated magnesium and a bioresorbable suture. Both implantable applications require highly biocompatible nanomaterials that do not cause any inflammation upon implantation.

In addition to implantable systems, nanomaterial-based wearable electronics also show great promise in human–machine interfaces, as exemplified by the triboelectric human–machine interface (THMI) nanophotonics system proposed by Bowei Dong and his collaborators shown in Figure 6h [112]. This system can convert certain human force signals, which cause deformation of the wearable devices, into an electrical signal via a triboelectrification and electrostatic induction process. Subsequently, a nanophotonic readout circuit translates this electrical signal into a photonic signal that can be used in human–machine interactions. The wearable devices are affixed to a glove worn on a human hand, and a robotic hand can mimic the gestures of a human hand.

**Figure 7:**
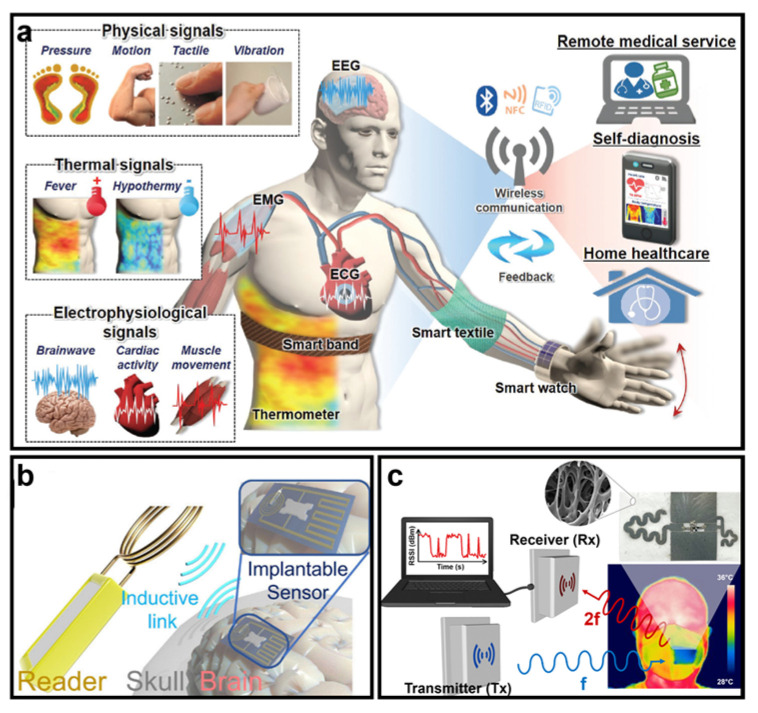
(**a**) A comprehensive diagram of wearable healthcare monitoring systems (reprinted with permission from Ref. [119], Copyright 2018 Royal Society of Chemistry). (**b**) Diagram of a wireless intracranial pressure monitoring system based on a nanomaterial-enabled piezoresistive sensor (reprinted with permission from Ref. [120], Copyright 2022 Institute of Electrical and Electronics Engineers). (**c**) Diagram of a smart face mask that can wirelessly monitor the mask-wearing condition and the cough frequency (reprinted with permission from Ref. [121], Copyright 2022 American Chemical Society).

Along these lines, the continuous, real-time, contactless, and accurate monitoring of biological signals of human bodies has become an attractive research area, with significant commercial potential due to its ability to provide invaluable information for post-surgery diagnosis, training plans for athletes, and self-health management. A comprehensive diagram of implementations of nanomaterial-based wearable electronics in healthcare monitoring is presented in Figure 7a, which includes physical indicators such as pressure, motion, tactile, vibration, and body temperature indicators for fitness plans and self-diagnosis, as well as vital signals such as intracranial pressure, ECG, and EMG information for clinical diagnosis and pre-/post-surgery treatments [119]. For example, Figure 7b depicts a wireless intracranial pressure monitoring system that implants a nanomaterial-based piezoresistive sensor beneath the cranium [120]. This sensor requires exceptionally high sensitivity and biocompatibility, as it interacts directly with the cerebrospinal fluid. Conventional methods for measuring intracranial pressure are highly dependent on wires or catheters and heavy-duty equipment, which cause discomfort and inconvenience for patients and may introduce risks such as inflammation or hemorrhage. In addition to vital sign monitoring, routine human activities are also critical. As demonstrated in Figure 7c, wearing masks during infectious disease pandemics, such as the coronavirus disease 2019 pandemic, is the most effective way to prevent the spread of disease [121]. Hence, monitoring whether masks are correctly worn and detecting cough frequency may be of great importance. This nanomaterial-enabled smart mask can wirelessly provide valuable information to track potentially contagious individuals and improve diagnostic accuracy. Such RF monitoring systems based on flexible nanomaterials may have a bright future in healthcare monitoring systems.

## 4. Outlook

In 2016, the global market for wearable electronics was valued at approximately USD 150 million and was expected to grow to USD 2.86 billion by 2025 [122]. We anticipate that medical applications will dominate most of the wearable market since healthcare has become one of the most significant problems concerning people in modern society. Although the market size of wearable electronics is now quite large and still rapidly growing, investigations into wearables may still be in their embryonic state. Large-scale implementation of wearable electronics still faces several urgent problems that could determine the direction of future investigations. First, the stability of wearables, especially for implantable devices, must be addressed, which highly relies on the development of nanomaterials with robust mechanical properties. Prolonged lifetime for implantable devices may significantly reduce the number of surgeries required to maintain such devices, thereby effectively avoiding the risks of unexpected complications. Secondly, to facilitate the implementation of wearable devices, they must be compact, lightweight, and biocompatible; therefore, biodegradable nanomaterials are highly sought-after in order to achieve noninvasive or minimally invasive monitoring. Furthermore, the sensitivity of wearable transducers is also critical, which requires the development of emerging nanomaterials that can achieve sensitive responses to different physical quantities. Additionally, wearable electronics are expected to be wireless and battery-less. In this vein, various physiological signs such as pressure, strain, vibration, temperature, ion concentration, glucose and blood oxygen levels, ECG, and EMG, among many others depicted in Figure 7a, can be contactlessly interrogated by mobile devices for diagnosis, and patients can have much more mobility and comfort during the medical course of treatment. We foresee that in the near future, wearable electronics will make breakthroughs with respect to the aforementioned challenges, which may further change and improve people’s lifestyles as the market size of wearables continues to expand.

## Figures and Tables

**Table 1:** Comparisons of the biocompatibility and stretchability of four nanoporous polymer-based flexible substrates.

Soft Substrate	Air Permeability	Waterproofness	Thermal Management	Stretchability	Pore Size
Porous SEBS [47]	High	Moderate	High	High	200–800 nm
Porous PE [48]	No	No	Low	High	50–1000 nm
Porous PDMS [52]	High	\	Moderate	Moderate	~500 nm
Porous PU [53]	High	Moderate	No	Moderate	\

**Table 2:** Comparisons of mechanical and electrical properties and fabrication difficulties of different nanomaterial-based conductors.

Soft Conductors	Flexibility	Stretchability	Toxicity	Fabrication Complexity/Cost	Conductivity
Ag NWs network [58]	High	High	\	Moderate/high	9190 S/cm
Ag NWs/PEDOT:PSS [59]	Moderate	Moderate	\	Moderate/high	1200 S/cm
Graphene oxide ink [60]	High	High	No	Easy/low	900 S/cm
CNT-PMPS-CCB [61]	High	High	No	Moderate/Moderate	248.8 S/m
Au nanomesh [62]	High	Moderate	No	Easy/high	18,867 S/cm
Porous liquid metal [63]	High	High	No	Easy/low	3.4 × 10^6^ S/cm
Molybdenum dioxide [64]	Moderate	Moderate	No	Difficult/low	11,363 S/cm

## Data Availability

Not applicable.
